# Predictors of weight stigma experienced by middle-older aged, general-practice patients with obesity in disadvantaged areas of Australia: a cross-sectional study

**DOI:** 10.1186/s12889-018-5556-9

**Published:** 2018-05-21

**Authors:** Catherine Spooner, Upali W. Jayasinghe, Nighat Faruqi, Nigel Stocks, Mark F. Harris

**Affiliations:** 1 UNSW Sydney, Centre for Primary Health Care and Equity, Sydney, NSW 2052 Australia; 2 University of Adelaide, Discipline of General Practice, 178 North Terrace, Adelaide, 5005 Australia

**Keywords:** Obesity, Stigma, Australia, Primary health care, Patients, Cross sectional study, Adults

## Abstract

**Background:**

Rates of obesity have increased globally and weight stigma is commonly experienced by people with obesity. Feeling stigmatised because of one’s weight can be a barrier to healthy eating, physical activity and to seeking help for weight management. The aim of this study was to identify predictors of perceived weight among middle-older aged patients with obesity attending general practices in socioeconomically disadvantaged urban areas of Australia.

**Methods:**

As part of a randomised clinical trial in Australia, telephone interviews were conducted with 120 patients from 17 general practices in socioeconomically disadvantaged of Sydney and Adelaide. Patients were aged 40–70 years with a BMI ≥ 30 kg/m^2^. The interviews included questions relating to socio-demographic variables (e.g. gender, language spoken at home), experiences of weight-related discrimination, and the Health Literacy Questionnaire (HLQ). Multi-level logistic regression data analysis was undertaken to examine predictors of recent experiences of weight-related discrimination (“weight stigma”).

**Results:**

The multi-level model showed that weight stigma was positively associated with obesity category 2 (BMI = 35 to < 40; OR 4.47 (95% CI 1.03 to 19.40)) and obesity category 3 (BMI = ≥ 40; OR 27.06 (95% CI 4.85 to 150.95)), not being employed (OR 7.70 (95% CI 2.17 to 27.25)), non-English speaking backgrounds (OR 5.74 (95% CI 1.35 to 24.45)) and negatively associated with the HLQ domain: ability to actively engage with healthcare providers (OR 0.12 (95% CI 0.05 to 0.28)). There was no association between weight stigma and gender, age, education or the other HLQ domains examined.

**Conclusions:**

Weight stigma disproportionately affected the patients with obesity most in need of support to manage their weight: those with more severe obesity, from non-English speaking backgrounds and who were not in employment. Additionally, those who had experienced weight stigma were less able to actively engage with healthcare providers further compounding their disadvantage. This suggests the need for a more proactive approach to identify weight stigma by healthcare providers. Addressing weight stigma at the individual, system and population levels is recommended.

**Trial registration:**

The trial was registered with the Australian Clinical Trials Registry ACTRN126400102162.

## Background

Obesity has become a global health issue, accounting for over four million deaths globally in 2013 [1]. Not only have global rates of obesity increased in the past 20 years, but it is the higher categories of obesity that are growing at the fastest rates [2, 3]. In Australia, the prevalence of obesity rose from 19 to 28% between 1995 and 2014–15 [4]. People in higher categories of obesity are at substantially increased health risk because mortality increases sharply as body mass index (BMI: kilograms/metres^2^) rises above 30 [5].

In addition to physical health risks, people with obesity commonly experience weight-related stigma (“weight stigma”) [6–8]. Stigma has been defined as: “the cooccurrence of labeling, stereotyping, separation, status loss, and discrimination in a context in which power is exercised” [9]. Link and Phelan categorised three types of weight stigma: 1) Direct (such as being abused when using public transport); 2) Environmental (such as not being able to fit into seats on aeroplanes); and 3) Indirect (such as people staring at the contents of their supermarket trolley) [10]. People with obesity experience all three forms of stigma, including discrimination in employment settings (e.g. not hired for a job), educational settings (e.g. discouragement from seeking higher education), from service providers (provision of poorer service), and in interpersonal relationships (e.g. being treated with less courtesy and respect than other people and/or being insulted) [11]. Research has further shown that people from various backgrounds regard people with obesity as lazy, unintelligent and lacking self-discipline [12, 13]. Health care providers have been included in those service providers who hold negative attitudes towards people with obesity [14]. For example, in an Australian study GPs expressed frustration with overweight patients because they were noncompliant and lacked motivation [15]. Interviews with people with obesity suggested that they internalised this stigma, agreed with the negative judgements of others, and felt shame and inferiority [6, 9].

Weight stigma has been found to have negative impacts on the mental health of people with obesity (e.g. depression, anxiety, low self-esteem and poor body image) [13, 16]. These negative impacts have a range of consequences that constitute barriers to weight loss. For example, systematic reviews have found evidence of weight stigma contributing to maladaptive eating behaviours [13] and maladaptive coping strategies (e.g., isolating oneself) [16]. Research has identified how people with obesity avoid situations where they think they will be stigmatised thus creating a barrier to health promotion or seeking health care [6, 14]. Longitudinal research has suggested that weight discrimination may lead to weight gain [17].

Understanding the sociodemographic, physical and psychosocial characteristics of those patients with obesity who are most likely to experience weight stigma would help identify those patients most in need of support. Puhl and colleagues analysed data from a nationally representative sample in the United States of America (USA). They identified that the experience of weight discrimination increased with BMI and was more likely among younger people and women. Race, education, marital status and occupation were not predictive of discrimination [11]. While socioeconomic factors were not significant in this study, other evidence suggests that disadvantage could exacerbate the effects of weight stigma. A meta-analysis of data from correlational studies of discrimination identified that perceived discrimination was more harmful for psychological well-being in disadvantaged groups than advantaged groups [18].

The health literacy of a person could also be relevant to weight stigma. Health literacy relates to “accessing, understanding and using information to make health decisions” [19]. Low health literacy has been associated with poorer health management behaviours and outcomes [20]. The World Health Organization (WHO) identified it as a key determinant of health and health inequalities, particularly for migrants [21]. If low health literacy among people with obesity is associated with weight stigma, this would form a type of double jeopardy in terms of feeling shame and limiting access to professional help to manage obesity. Previous research has identified that people with low health literacy feel shame about their literacy level [22–24]. Most studies of the relationship between health literacy and stigma investigate how the health literacy of a population affects the likelihood that they will stigmatise a particular group, and most of this research relates to mental health [25–27]. However, we could not identify literature on a relationship between health literacy and perceived stigma among patients with obesity. The only identified study examining the relationship between health literacy and perceived stigma within a group living with a stigmatised health condition involved people with epilepsy [28]. This study identified that the experience of stigma among people with epilepsy was associated with low health literacy, as indicated by difficulties understanding written information.

There is a gap in our knowledge regarding how weight stigma might vary with obesity category, sociodemographic factors and health literacy. Such information can assist health-care and public-health practitioners to focus their interventions appropriately. We tested the hypothesis that perceived weight stigma among patients with obesity was associated with level of obesity, demographic variables (sex, language spoken, employment, education and age) and health literacy.

## Methods

### Aim

The aim of this study was to identify predictors of perceived weight stigma (as indicated by perceived ridicule and discrimination due to weight) among patients with obesity attending general practices in socioeconomically disadvantaged urban areas of Australia. The predictors investigated were sociodemographic factors (age, sex, language spoken at home, education level and occupation), obesity category and health literacy.

### Study design

Cross-sectional data from telephone interviews with patients with obesity were used in this analysis. The interviews were part of the baseline data collection for a pragmatic cluster randomised trial of an intervention that aimed to improve the management of obesity in general practices. The study protocol has been published elsewhere so methods will only be summarised here [29].

### Setting

The study was conducted in 2015. Patients were recruited from 17 general practices in socioeconomically disadvantaged areas of Sydney and Adelaide that had agreed to participate in a study of weight management in patients with obesity from disadvantaged background with low health literacy. Initially 20 practices had been recruited to the study, but three dropped out (one in Sydney and two in Adelaide).

### Participants

The study inclusion criteria were: age 40–70 years, BMI≥30, attended the practice at least once in the previous 12 months, no history of chronic disease (heart disease, insulin-treated diabetes, chronic renal impairment) or stroke, no current treatment with a weight loss medication, and no previous or planned bariatric surgery. Eligible patients were given a package that included information about the study and a consent form. After patients had completed the screening questions, their general practitioner (GP) checked their eligibility for the study. Initially, patients were excluded if categorised as having adequate health literacy by a health literacy screening questionnaire [30]. However, this was abandoned because of poor correlation between the screening instrument and the HLQ. Consequently, 43% of the sample had adequate health literacy according to the screening questionnaire. This rate is consistent with an Australian population survey of health literacy, which showed that 40% of adults had adequate health literacy [31]. Patients also needed to be able to read and write in English, Arabic, Chinese, Hindi, or Italian as the package was only available in these languages. Out of 204 patients who consented to participate, 41 were excluded as they failed to match the eligibility criteria, withdrew or commenced the intervention before completing the baseline data collection. Of the 163 enrolled, 120 patients completed the interview.

### Variables

The outcome variable was perceived weight stigma in the previous week (yes or no). Predictor variables were obesity category (1, 2 or 3, defined below), sex (male or female), language background (English or non-English language mostly spoken at home), employment (employed or not employed), highest qualification (school or higher qualifications), age (40–56 or 57–70 years) and health literacy. Obesity category was calculated using the WHO classification for obesity: Category 1 BMI = 30–34.99; Category 2 BMI = 35–39.99, Category 3 BMI ≥40. The category “not employed” included unemployed and looking for work, retired, home duties, or unable to work due to long-term sickness or disability.

### Instruments

Perceived weight stigma was measured using two items from *The Impact of Weight on Quality of Life-Lite Measure* (IWQOL-Lite). This is a validated self-report measure of obesity-specific quality of life for adults in the previous week [32, 33]. It scores five domains, one of which is public distress, which comprises five items. Three of these items relate to environmental forms of discrimination (e.g. worry about fitting into seats), whereas two relate to direct stigma. These latter are:

- Because of my weight I experience ridicule, teasing, or unwanted attention.
- Because of my weight I experience discrimination by others.

These two forms of direct stigma were combined into a categorical variable to indicate the experience of direct stigma in the previous week: 0 = Both not true; 1 = At least one true.

BMI was calculated using weight and height measurements taken by the GP at the time of recruitment. Socio-demographic variables were measured using questions used in our previous research.

Health literacy was assessed using the Health Literacy Questionnaire (HLQ), which provides scores on nine domains (Table 1) [34]. Each of the nine scales has demonstrated reliability with composite reliability scores ranging from 0.8 to 0.9 [35]. Respondents rated how much they agreed or disagreed with statements relating to domains 1–5 (response scale 1–4); and how difficult or easy they found tasks relating to domains 6–9 (response scale 1–5).

**Table 1:** Domains of the Health Literacy Questionnaire

Domain abbreviation	Domain
1. HPS	Feel understood and Supported by Healthcare Providers
2. HSI	Have Sufficient Information to manage my health
3. AMH	Actively managing health
4. SS	Have Social Support for health
5. CA	Critically Appraise health information
6. AE	Ability to Actively Engage with healthcare providers
7. NHS	Navigating the Healthcare System
8. FHI	Ability to Find good Health Information
9. UHI	Ability to Understand Health Information well enough to know what to do

### Data collection

The data reported here were collected by trained research staff via telephone interview in English, Arabic or Italian.

### Data analysis

Univariate analyses (chi-square tests of linear-by-linear association, independent samples test) were initially conducted using SPSS statistical software (version 22) to describe the distributions of individual variables, identify bi-variate associations and check for collinearity of variables.

Multilevel logistic regression models were used with the dichotomous dependent variable stigma adjusted for clustering of patients (level 1) within general practices (level 2) [36]. Initially, we fitted the full model followed by the final model. The significant (*P* < 0.05) independent variables in the univariate analyses were included in the full model subject to multicollinearity (a state of very high intercorrelations among the independent variables). We found that NHS (Navigating the Healthcare System) was highly correlated with AE (Active Engagement) (*r* = 0.713) and FHI (Find Health Information) (*r* = 0.752). Similarly, UHI (Understand Health Information) was highly correlated with FHI (*r* = 0.775). The general approach for dealing with multicollinearity involves inclusion of one of the two highly correlated predictor variables [37]. To avoid the multicollinearity, we did not include NHS (Navigating the Healthcare System) and UHI in our multivariate analysis. The significance of the independent variables was assessed using the Wald joint χ2 test statistic [36]. There were no meaningful differences in the results of the full model and the final model. Only the significant independent variables in the full model were included in the final model. Therefore, those for the final model were discussed in this paper.

The intracluster correlation coefficient (ICC) was calculated using the latent variable method. The (standard) logistic distribution has variance π^2^/3 = 3.29 and hence this can be taken as the level 1 variance. As both the level 1 and 2 variances are on the same scale, the following formula was used: ICC = (level 2 variance)/(level 2 variance + 3.29) [38]. All multi-level models were performed with MLwiN version 2.30 [36].

## Results

### Participants

Participant and practice (location and size) characteristics are presented in Table 2. About one-third of the participants spoke a language other than English at home and two-thirds were female. Age (40–56 and 57–70 years) and qualifications (none/school or a higher qualification) were dichotomised. About half were in obesity Category 1, while the other half were in Category 2 or 3. Just over half attended a small general practice (1–3 GPs) and slightly more lived in Sydney than in Adelaide. About one-third had experienced direct weight-related stigma in the previous week.

**Table 2:** Patient and provider (practice) characteristics

Variable	Number	Category	Number	Percent
Patients				
Sex	120	Female	78	65
		Male	42	35
Age	120	40–56	65	54
		57–70	55	46
Language mostly spoken at home	119	English	81	68
		Non-English language^a^	38	32
Employment	119	Employed	58	49
		Not employed^b^	61	51
Highest qualification	119	School^c^	57	48
		Higher qualifications^d^	62	52
Obesity category	117	1 (BMI = 30–34.99)	54	46
		2 (BMI = 35–39.99)	40	34
		3 (BMI ≥ 40)	23	20
Experienced stigma in past week	118	Yes	38	32
		No	80	68
Providers				
Practice size	120	1–3 GPs	52	43
		4+ GPs	68	57
City	120	Adelaide	51	43
		Sydney	69	57

^a^The most commonly spoken languages at home were Arabic (*n* = 11), Spanish (*n* = 7) and Italian (*n* = 5)

^b^Very few of those who were not employed were unemployed and looking for work (4%). The remainder were mostly retired (22%), home duties (13%), or unable to work due to long-term sickness or disability (9%)

^c^“School” = no formal education, or primary or high school certificate

^d^“Higher qualifications” = Technical and Further Education (TAFE) qualification or University degree

### Predictors of stigma

#### Univariate analyses

Univariate analyses are presented in Table 3. Patients who reported weight stigma were significantly *more* likely than other patients to be in a higher obesity category, speak a non-English language at home, not employed and to have a lower score on seven of the nine HLQ domains.

**Table 3:** Univariate associations with stigma

Patient variable	No Stigma %	Stigma %	ChiSq	DF	*p*
Obesity Category 1	54	28	10.382	1	*.001* ^*a*^
Obesity Category 2	33	36			
Obesity Category 3	13	36			
Sex = Male	35	34	0.007	1	.993
Non-English Language	23	50	9.052	1	*.003*
Not working	39	79	16.670	1	*.000*
Highest qualification = School	46	50	0.145	1	.703
Age group = 57–70 years	48	45	0.079	1	.779
Patient health literacy	No Stigma (Mean)	Stigma (Mean)	t	df	*p*
1. HPS	3.2	3.1	−1.164	114	.247
2. HSI	3.0	2.6	−3.967	114	*.000*
3. AMH	2.7	2.5	−2.429	114	*.017*
4. SS	3.1	2.8	−2.788	115	*.006*
5. CA	2.8	2.6	−1.295	114	.198
6. AE	4.2	3.7	−3.910	114	*.000*
7. NHS	3.9	3.3	−3.867	114	*.000*
8. FHI	3.8	3.3	−2.975	113	*.004*
9. UHI	4.0	3.6	−3.011	115	*.003*
Practice variable	No Stigma %	Stigma %	ChiSq	DF	*p*
City = Sydney	54	66	1.5	1	.218
Practice size = 1–3 GPs	45	40	.318	1	.573

All *p* values for Pearson’s chi-square except ^a^ (^a^ = chi-square for linear-by-linear association). Note: Significant associations are given in italics

### Multi-level analysis

Perceived weight stigma was positively associated with obesity category 2 (BMI = 35–39.99; OR 4.47 (95% CI 1.03 to 19.40)) and obesity category 3 (BMI = ≥ 40; OR 27.06 (95% CI 4.85 to 150.95)), not being employed (OR 7.70 (95% CI 2.17 to 27.25)) and non-English language spoken at home (OR 5.74 (95% CI 1.35 to 24.45)). It was negatively associated with one HLQ domain: ability to actively engage with healthcare providers (OR 0.12 (95% CI 0.05 to 0.28)) (Table 4). There was no association between perceived weight stigma and gender, age, education or the other HLQ domains in the univariate analysis.

**Table 4:** Multi-level logistic regression models for stigma

Explanatory Variables (reference)	Full Model 1	Final Model 2
Patient factors	OR (95% CI)	OR (95% CI)
BMI (kg/m^2^)	1.00 (reference)	1.00 (reference)
35–39 (30–34)	**5.23 (0.92–29.86)**	**4.47 (1.03–19.40)**
≥ 40 (30–34)	**34.99 (2.55–479.89)**	**27.06 (4.85–150.95)**
Employment	1.00 (reference)	1.00 (reference)
Not employed (employed)	**9.89 (2.06–47.56)**	**7.70 (2.17–27.25)**
Language spoken at home	1.00 (reference)	1.00 (reference)
Non-English (English)	**6.86 (1.39–33.83)**	**5.74 (1.35–24.45)**
Health literacy domains (continuous variables)		
AMH: Actively managing health	1.60 (0.25–10.10)	
SS: Have social support for health	0.68 (0.14–3.26)	
HIS: Sufficient Information to manage health	0.24 (0.03–1.81)	
AE: Actively engage with healthcare providers	**0.18 (0.06–0.56)**	**0.12 (0.05–0.28)**
FHI: Ability to Find good Health Information	1.42 (0.48–4.15)	
Intra-cluster correlation (ICC)	0.326	0.255

Note: Significant estimates are given in bold text

## Discussion

The purpose of this study was to test whether perceived weight stigma among patients with obesity was associated with level of obesity, demographic variables and/or health literacy. We found that one-third of the sample had experienced direct forms of weight discrimination in the week before being interviewed. Weight discrimination was more likely to be experienced by patients in higher obesity categories, who were not employed, who spoke a language other than English at home and who had lower scores on the HLQ domain that measures the ability to actively engage with healthcare providers (AE).

The increased likelihood of experiencing weight discrimination as obesity category increased was consistent with research conducted in the USA [11]. It is not possible, however, to compare the size of the relationship with the USA research as their base for comparison was normal weight, whereas ours was obesity category 1. The rising prevalence of the higher categories of obesity suggests that weight discrimination will be an increasing problem. This highlights the need for strategies not only to reduce obesity, but also to address weight discrimination and to build the resilience of patients with obesity.

There is little comparable research on how socio-demographic factors relate to weight discrimination. Puhl and colleagues’ study in the USA investigated these factors using data from a community sample [11]. The relationships between weight discrimination and age, sex, occupation, education and cultural background in our study were not consistent with Puhl et al’s study. In that study, age and sex were significant predictors of weight discrimination and they found no independent effect of race or education on the likelihood of weight discrimination. Many differences between the studies could explain the different results, including Puhl et al’s greater power to detect an effect (*N* = 2290), focus on gender analyses, the broader age range of the sample (theirs was 25–74 years), different setting (communities in the USA) and different measures of weight discrimination. It is only with multiple studies across settings and population groups that patterns might be clearly identified. Our finding that weight discrimination was significantly more likely for patients who spoke a non-English language at home and who were not in the workforce, independent of BMI, suggests that normative and cultural social factors may contribute to stigma. These are vulnerable groups on many indicators. Obesity stigma exacerbates their disadvantage.

No previous research on the relationship between weight stigma and health literacy was identified, so we cannot compare these results with other research. Patients who had experienced weight discrimination in the previous week had lower mean scores on all nine of the HLQ scales and these differences were significant for seven of the scales in the univariate analyses. Only active engagement with health care providers (AE) was significant in multi-level analysis after adjustment for confounding factors and cluster effects (ICC > 0.25). Bautista et al. found a relationship between health literacy and stigma among patients with epilepsy [28]. While there are many differences between their study and ours, both suggest a relationship between health literacy and a stigmatised health condition. The relationship between weight stigma and health literacy might be due to their shared risk factors: socio-economic status and speaking a language other than English [39, 40].

People with low scores in the AE (Active Engagement) domain tend not to consider their health as being their own responsibility and to be not engaged with their healthcare [34]. Previous research has shown that the experience of weight discrimination can be a barrier to participation in health-promoting behaviours and a reluctance to seek help and support [6]. The finding from our study suggests that patients experiencing weight stigma may also experience barriers to engagement with health care providers – a potential source of support. Active engagement strategies by PHC providers will be particularly important for those patients with obesity and experiencing discrimination.

This study is consistent with observations that weight discrimination is experienced by patients with obesity, and that it is more likely for those groups who already experience other forms of discrimination: those who speak a language other than English and those who are not in the workforce. It is also more prevalent among those who are not actively managing their own health. Given the evidence that health practitioners are a source of weight stigma, [14] there is a need for interventions to develop awareness of health professionals in how they could be contributing to perceived weight stigma [41].

There are many ways in which health professionals can support patients with obesity who are experiencing weight discrimination. For example, words that may be considered offensive to patients with obesity should be avoided [42]. Health professionals could discuss obesity stigma and its effect on patient’s lives, tailor interventions to match patients’ preferences (e.g. identify exercise programs that will not expose the patient to further stigma), ensure the practice is not stigmatising (e.g. provide chairs that are big enough for patients with obesity), and demonstrate empathy for their experience [43]. Phelan et al.’s review identified a number of interventions that could potentially reduce the impact of obesity stigma on the provision of health care [14]. These included educating health professionals about the complex factors that contribute to obesity so that they have a better understanding of the difficulties faced by patients in weight loss. Mittal et al. reviewed interventions for patients and concluded that interventions that enhance the coping skills of people who experience stigma (e.g. build self-esteem, develop help-seeking behaviour) show promise [43]. However, addressing stigma is challenging, [13] and it is likely that no single intervention will be effective.

This study had a number of limitations. Patients with obesity were recruited from general practices so results cannot be generalised to people with obesity in the general community. The sample size was small so the power to detect relationships was low, but the data were robust as validated instruments were used for data collection. The cross-sectional design did not enable the direction of any of the relationships to be investigated. However, this research was the first study to investigate the relationships between weight discrimination, obesity category, socio-demographic variables and health literacy. There is a need for further research on the predictors of perceived weight stigma in different populations and how perceived weight stigma impacts upon seeking and engaging with primary health care services that can support weight management.

## Conclusions

Weight stigma disproportionately affects the patients with obesity most in need of support to manage their weight: patients whose obesity is category 2 or above, patients from non-English speaking backgrounds and patients not in the workforce. The ability to actively engage with healthcare providers was negatively associated with the likelihood of weight stigma. These trends suggest that weight stigma may compound other forms of social disadvantage. Strategies are needed to address weight stigma at the individual, system and population levels and to educate primary care providers to be more alert to the needs of their patients with obesity.

## Abbreviations

BMI: Body mass index
GP: General Practitioner
HLQ: Health Literacy Questionnaire
ICC: Intracluster correlation coefficient
IWQOL-Lite: Impact of Weight on Quality of Life-Lite Measure
OR: Odds ratio
USA: United States of America

## HLQ domains

HPS: Feel understood and Supported by Healthcare Providers
HSI: Have Sufficient Information to manage my health
AMH: Actively managing health
SS: Have Social Support for health
CA: Critically Appraise health information
AE: Ability to Actively Engage with healthcare providers
NHS: Navigating the Healthcare System
FHI: Ability to Find good Health Information
UHI: Ability to Understand Health Information well enough to know what to do
